# Nonlinear Decoupling Study of Six-Axis Acceleration Sensor Based on Improved BP Neural Network

**DOI:** 10.3390/s25072280

**Published:** 2025-04-03

**Authors:** Jialin Zhang, Chunzhan Yu, Chengxin Du, Zhe Hao, Zhibo Sun

**Affiliations:** 1 The School of Technology, Beijing Forestry University, Beijing 100083, China; zhangjialin@bjfu.edu.cn (J.Z.); chengxindu97@gmail.com (C.D.); 18612864280@163.com (Z.H.); 2 Beihang School, Beihang University, Beijing 100191, China; sunzb@buaa.edu.cn

**Keywords:** six-axis accelerometer, parallel mechanism, improved BP neural network, gradient descent with momentum, nonlinear decoupling

## Abstract

Aiming at the problem of nonlinear coupling error in the measurement of parallel six-axis accelerometers, this study improves the back propagation (BP) neural network and proposes an improved BP neural network decoupling model that introduces the gradient descent with momentum and the Levenberg–Marquardt (LM) algorithm. By introducing the momentum factor in the model updating stage, the LM algorithm is used in the local learning stage to improve the convergence speed and shock resistance of the network, and to enhance the accuracy of the algorithm. Based on the mid-frequency standard vibration device APS 129 ELECTRO-SEIS (SPEKTRA, Stuttgart, Baden-Württemberg, Germany), the calibration data are obtained and the improved BP neural network decoupling model is trained to complete the nonlinear decoupling of the test set. Compared with the linear decoupling method, the decoupled six-axis accelerometers with the improved BP neural network model have acceleration measurement accuracies of 0.035%, 0.018% and 0.039% along the x, y and z axes, respectively, which indicates that the model has high decoupling accuracy, and it can significantly improve the measurement accuracy of the sensors. The research results can provide theoretical support for high-precision inertial navigation.

## 1. Introduction

The six-axis acceleration sensor is a crucial component for measuring the motion parameters of an object in six axes. It is capable of detecting the object’s linear acceleration along three axes and its angular acceleration along three orthogonal axes in space. It is widely utilized in the high-precision acquisition of carrier position data, particularly in the field of intelligent robot control [1], inertial navigation system [2], vision system anti-jitter [3], touch mobile device attitude detection [4], human health detection and localization rescue [5,6,7], and biomedical treatment [8,9]. Compared with the traditional inertial navigation module, the parallel six-axis accelerometer has a strong anti-disturbance ability and a smaller cumulative error [10], which can provide a guarantee for the accuracy of the object’s positional information. Additionally, the parallel structure principle allows for the integration and simultaneous output of holographic inertial information, facilitating the integration of inertial sensing devices.

However, due to the elastic element structure of the six-axis accelerometer, machining and assembly errors, detection circuit noise and other aspects of the six-axis accelerometer, the six-axis accelerometer signal output of the various paths presents strong coupling, nonlinear characteristics and the existence of nonlinear coupling error seriously affects the sensor’s measurement accuracy [11,12], which restricts the application of the parallel-type six-axis accelerometer in the high-precision inertial navigation system. At the same time, the mechanism behind these nonlinear coupling errors is complex and difficult to describe quantitatively. As a result, developing a high-precision decoupling method capable of inhibiting or even eliminating these coupling errors has become a critical challenge for the engineering application of parallel six-axis accelerometers.

In order to solve the inter-dimensional coupling problem of multidimensional sensors, high-precision decoupling methods are proposed, and the current research is categorized into two main groups. One type is to start from the research of the design principle and manufacturing process and try to eliminate the root cause of its generation [13,14,15]. The other is to study the suitable decoupling algorithm to reduce the accelerometer coupling error by numerical operation. However, the first method is difficult to construct, which is not conducive to the popularization of the application. Yao et al. [16] used the least-squares method to decouple the three-dimensional force sensors, and the maximum error of the three orthogonal directions in the space can be controlled at 0.8%. Feng et al. [17] added a nonlinear error term based on polynomial fitting for the decoupling model on the basis of the linear decoupling method, which significantly improves the accuracy of the decoupling algorithm compared to linear decoupling algorithms. Liang et al. [18] used extreme learning machine for nonlinear decoupling; this algorithm has obvious advantages in training speed and avoiding local optimums, but it is more sensitive to noisy data and the training results have a certain degree of instability with the selection of initialization parameters [19]. Qian et al. [20] used a standard back propagation (BP) neural network for nonlinear decoupling of multidimensional sensors, and the results showed that the standard BP neural network decoupling accuracy is better, but there are shortcomings such as insufficient global convergence ability and slower convergence speed.

In this paper, a nonlinear decoupling algorithm for a six-axis accelerometer based on improved BP neural network is proposed for the nonlinear and strong coupling problems existing in the current parallel six-axis accelerometer measurement research, as well as the shortcomings of the standard BP neural network. The decoupling accuracy and convergence speed are improved by introducing the gradient descent algorithm with momentum and the Levenberg–Marquardt algorithm (LM), and the model training is completed based on the calibration data obtained from standard loading experiments. The feasibility of the algorithm is verified by comparing with the linear decoupling method, which provides a reference for the study of high-precision nonlinear decoupling of the six-axis accelerometers.

## 2. Six-Axis Accelerometer Decoupling Principle

**Figure 1:**
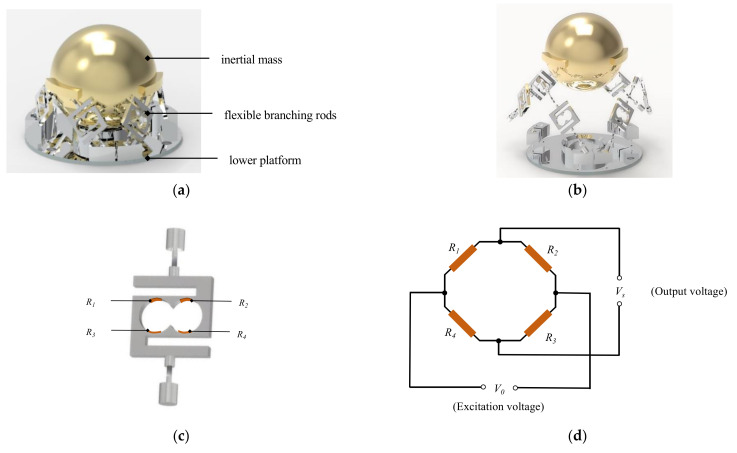
Six-axis accelerometer based on Gough–Stewart Platform: (**a**) the structure of the six-axis accelerometer; (**b**) exploded view of the six-axis accelerometer; (**c**) position of strain gauges on branch rods; (**d**) measuring circuit for branch rods output voltage.

The accelerometer studied in this paper is a parallel six-axis accelerometer with the measurement principle of resistive strain, and the overall structure is shown in Figure 1, which consists of an inertial mass block, six elastic branching rods and a lower platform.

According to the Gough–Stewart constitutive principle, there are six branching rods between the mass block and the lower platform. When acceleration is applied to the six-axis accelerometer, a small relative displacement tends to occur between the mass block and the lower platform, and the six branching rods are stressed to produce strain. Strain gauges are affixed to specific sensitive areas of the branch rods shown in Figure 1 to measure the magnitude and direction of the strain, and the strain signals are converted to analog voltage signals by a Wheatstone full-bridge circuit. In order to improve the signal-to-noise ratio of the sensor, the mass block is made of brass, and the sensitive areas of the branch rods are designed with rounded skeletons. The main structure of the sensor is CNC machined and assembled with threaded connections.

When the accelerometer is subjected to an acceleration load, the output voltage signal of the measurement circuit already contains the errors caused by inter-dimensional coupling and other factors. Therefore, a decoupling operation must be performed on the sensor. The output voltage signal matrix **V** and the corresponding input acceleration load vector matrix ***α*** are obtained after multiple loading of the six-axis accelerometer, and the following expression for the input–output relationship can be established:

(1) α=CV

where ***α*** is the acceleration load vector obtained by multiple loading along the direction of each degree of freedom; **V** is the output voltage signal of each channel corresponding to the acceleration load vector of multiple loading. **C** is the mapping relationship matrix between the input acceleration load vector and the output voltage signal. ***α*** is in the form shown in Equation (2):

(2) $$\alpha =\left[\begin{array}{ccc} \begin{array}{cc} a_{x1} & a_{x2}\\ a_{y1} & a_{y2} \end{array} & \cdots & \begin{array}{c} a_{xn}\\ a_{yn} \end{array}\\ \vdots & \ddots & \vdots \\ \begin{array}{cc} \varepsilon_{z1} & \varepsilon_{z2} \end{array} & \cdots & \varepsilon_{zn} \end{array}\right]$$

where $a_{ij}$ is the linear acceleration for multiple loadings along the orthogonal axial direction in space and $\varepsilon_{ij}$ is the angular acceleration for multiple loadings in the direction of rotation around the orthogonal axial direction in space.

Since the signals output from the strain gauges are analog voltage signals rather than the physical quantity of acceleration to be measured, the correspondence between the acceleration input signals and the sensor output electrical signals, i.e., the mapping relationship matrix C, obtained through calibration, determines the accuracy of the final acceleration information obtained. Therefore, obtaining the mapping relation matrix with smaller error is the key to improve the decoupling accuracy of the six-axis accelerometer.

## 3. Nonlinear Decoupling Methods

### 3.1. Nonlinear Decoupling Model Based on BP Neural Network

BP neural network is a multi-layer forward neural network based on the error back propagation algorithm for learning and training. In the training process, through the back propagation of error signals from the back to the front, layer by layer, the weights of the network are corrected after a cycle of iteration until the output value of the network is in a stable state. To establish the BP neural network model and determine the mapping relationship between the input and output of the BP neural network model, the connection parameters between the input layer and the hidden layer and the hidden layer and the output layer must be obtained through sample training. The whole process includes two parts: forward propagation of the input signal and back propagation of the error signal.

To decouple the six-axis accelerometer using a BP neural network, the first step is to establish a BP neural network decoupling model. The six-axis accelerometer can measure the linear acceleration in the three orthogonal axes in space and the angular acceleration around the three orthogonal axes. Therefore, both the input vector α and output vector V have a dimension of n=6. The output vector V of the six-axis accelerometer is the input vector of the neural network decoupling model.

**Figure 2:**
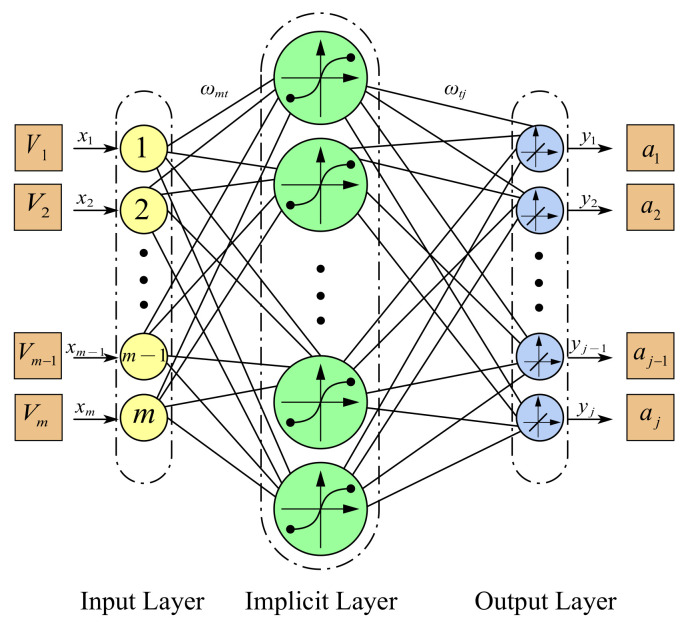
Topology of neural networks.

Based on the parallel six-axis accelerometer studied in this paper, a BP neural network topology containing one hidden layer is used. The input layer of the network is the output voltage signal of the six-axis accelerometer, and the output layer of the network is the acceleration-to-be-measured signal obtained by decoupling the model. The number of neurons in the input layer of the neural network $n_{i}=6$. The number of neurons in the output layer $n_{0}=6$. The number of neurons in the hidden layer is determined according to the empirical formula $n_{h}=\sqrt{n_{i+n_{0}}}+x$ (where x is an integer between 1 and 10). In order to make the output of the neural network present a nonlinear characteristic, the nonlinear and microscopic Sigmoid function is chosen as the activation function between the input and hidden layers. Among the different types of Sigmoid functions, the Tan-Sigmoid function with a large output range is chosen: $f\left(x\right)=\frac{e^{x}-e^{-x}}{e^{x}+e^{-x}}$. A linear function l is used as the transfer function between the hidden layer and the output layer. The established decoupling model of BP neural network is shown in Figure 2.

The output of the BP neural network decoupling model is:

(3) $$T=l[\sum_{k=1}^{n_{h}}S(\sum_{i=1}^{n_{0}}P_{i}\omega_{ik})\omega_{kj}]$$

where i=1,2,⋯,6; $k=1,2,\cdots ,n_{h}$; $P_{i}$ is the neural network input value; T is the neural network output; S(⋯) and l(⋯) are the hidden layer activation function and the output layer activation function, respectively; $\omega_{ik}$ is the connection weight between the *i*-th neuron of the input layer and the *k*-th neuron of the hidden layer; and $\omega_{kj}$ is the connection weight between the *k*-th neuron of the hidden layer and the *j*-th neuron of the output layer.

### 3.2. Global Training Function Optimization Based on Gradient Descent with Momentum

The gradient descent algorithm in BP neural networks is mainly used to optimize the model parameters in the back propagation process. While the prediction results of the BP neural network model based on the standard fastest algorithm are generally satisfactory, there are shortcomings such as a large number of iterations, slow convergence speed and violent network oscillation. To address these issues, the gradient descent algorithm with momentum is used for global training, while the Levenberg–Marquardt (LM) algorithm is applied for local learning in the standard BP neural network.

In the weight update stage of the standard BP neural network most-rapid descent method, the momentum factor α (0<α<1) is introduced to make the weight correction of the neural network have some inertia, as shown in Equation (4):

(4) $$\Delta \omega \left(n\right)=-\eta \left(1-a\right)\nabla e\left(n\right)+\alpha \Delta \omega (n-1)$$

After the introduction of the new momentum factor, if the direction of the gradient obtained from two adjacent iterations is the same, the momentum factor can enlarge the update amount of the weights to accelerate the convergence process so that the neural network can speed up the computation. In contrast, the update amount of the weights will be partially offset by the momentum factor so that the neural network has a certain degree of shock resistance, and it is easier to find the point of the minimum value.

### 3.3. Local Learning Function Optimization Based on LM Algorithm

The LM algorithm is an optimization algorithm based on Taylor expansion that corrects the weights of the neural network according to Equation (5):

(5) $$\omega \left(n\right)=\omega \left(n-1\right)-{\left(J^{T}J+\mu I\right)}^{-1}J^{T}e$$

where J is the Jacobi matrix of the first-order derivatives of the error function with respect to the network weights, $J^{T}e$ denotes the gradient, and, for a binary differentiable function f(x,y), $J^{T}J$ can be expressed as:

(6) $$J^{T}J=\left[\begin{array}{cc} \frac{\partial^{2}f}{\partial x^{2}} & \frac{\partial^{2}f}{\partial y\partial x}\\ \frac{\partial^{2}f}{\partial y\partial x} & \frac{\partial^{2}f}{\partial y^{2}} \end{array}\right]$$

The LM algorithm only requires knowledge of the gradient of the objective function and iterates by calculating the change in the gradient, and this method has a faster convergence rate when using scientific computing tools for the calculation.

Using the LM algorithm as well as the gradient descent with momentum as the global training function and the local learning function of the standard BP neural network, respectively, can make the neural network nonlinear decoupling model have better shock resistance while converging faster, reduce the coupling error of the outputs, and achieve the purpose of significantly improving the measurement accuracy of the sensor.

## 4. Decoupling Experiment and Result Analysis

**Figure 3:**
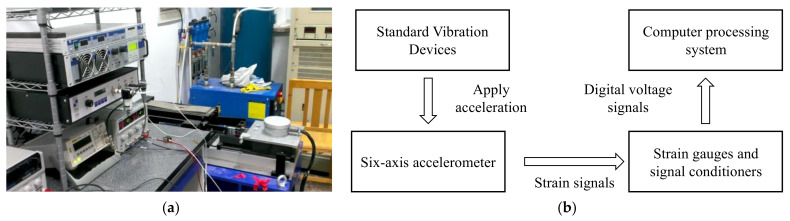
Static calibration system: (**a**) standard vibrating platform; (**b**) calibration test system.

The six-axis accelerometer studied in this paper was calibrated and sensitivity linear calibration experiments were carried out at the China Academy of Measurement Sciences. The comparative method medium-frequency vibration standard device APS 129 ELECTRO-SEIS used for the calibration platform and the experimental system are shown in Figure 3.

**Figure 4:**
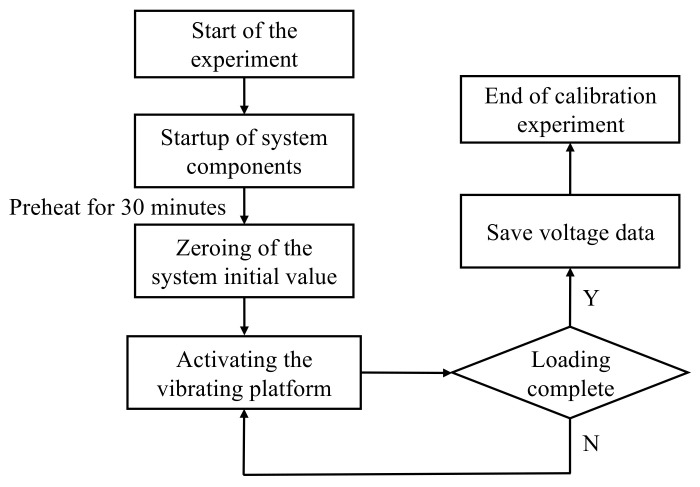
Calibration experiment flow.

The data required for neural network training were collected through calibration experiments. The flow of the calibration experiment is shown in Figure 4. Before starting the experiment, all system components were activated and warmed up for 30 min. After zeroing the system’s initial values, acceleration samples were set at equal intervals within the accelerometer’s range. The standard vibration platform was then initiated and the accelerometer was subjected to a sinusoidal acceleration signal. The output voltage of the accelerometer was recorded at the specified sampling points. During the experiment, the frequency of the sinusoidal acceleration signal input from the standard vibration platform was 16 Hz. The data acquisition system used in this experiment mainly consists of the signal acquisition instrument of model Inv3062c (China Orient Institute of Noise & Vibration Technology, Beijing, China) and the acquisition software on PC. The computer processing system sampled the output voltage at a frequency of 500 Hz and recorded the output voltage at equally spaced acceleration sampling points within the range.

The six-axis accelerometer was connected to the data acquisition system through a multichannel dynamic strain gauge. The output signals were collected by the data acquisition system on the computer. Through calibration experiments, we obtained six-channel digital voltages from the accelerometer output under equally spaced acceleration inputs. Part of the data of the calibration experiments are shown in Table 1, Table 2 and Table 3.

**Figure 5:**
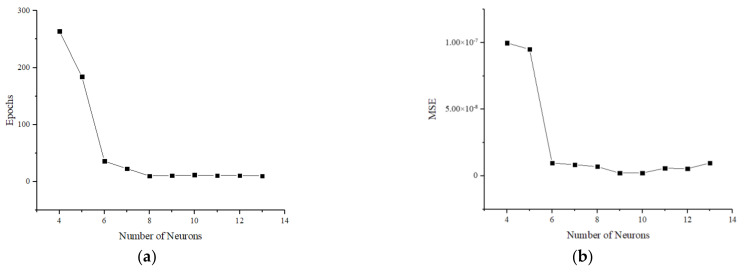
Relationship between neural network performance and the number of neurons in the hidden layer: (**a**) number of iterations; (**b**) MSE.

**Figure 6:**
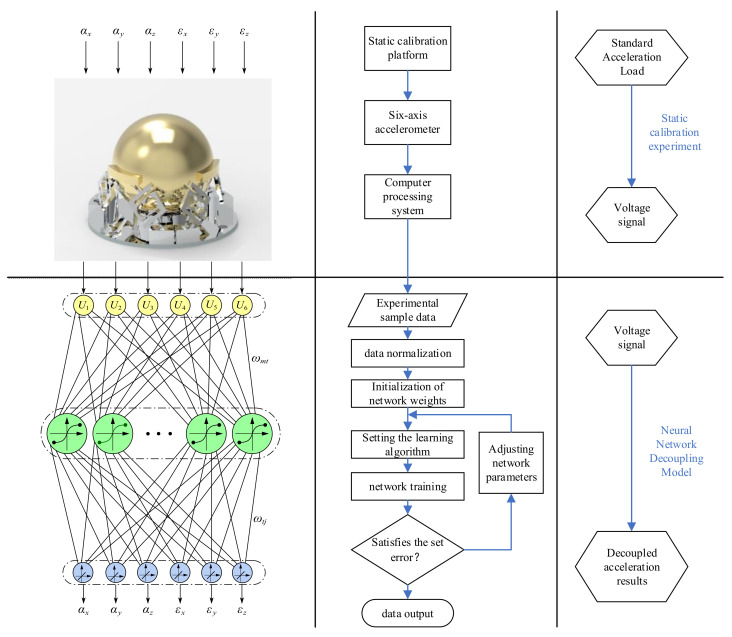
Decoupled calibration process for six-axis accelerometers.

The gradient descent with driving momentum is introduced into the BP neural network to establish a nonlinear decoupling model for the six-axis accelerometer with improved BP neural network. The data collected from the calibration experiments are used to train the neural network employing the LM algorithm. The maximum number of iterations in the training process is set to be 1000, the learning rate is 0.01 and the sum of target errors is 1 × 10^−5^. As shown in Figure 5, the number of network iterations and the mean square error of the decoupling results decrease rapidly as the number of hidden-layer neurons increases. The number of iterations reaches a minimum at 8 for the number of hidden layer neurons, while the mean square error reaches a minimum at 9 for the number of hidden layer neurons. In order to obtain the best decoupling accuracy, the number of neurons in the hidden layer is chosen to be 9. The specific calibration, training and decoupling processes are shown in Figure 6.

To improve the training effectiveness of the network and verify the decoupling accuracy of the network, the static calibration data are divided into two parts: the training set and the test set. The training set comprises 90% of the data, while the test set accounts for 10%. The model after the completion of training uses the voltage of the test set as the input, and the output of the model is the decoupled acceleration value. The data of the training results are shown in Table 4.

Using the trained improved BP neural network for nonlinear decoupling of the six-axis accelerometer, the decoupled value of acceleration output from each channel is almost the same as the loaded standard value. The linearity of the decoupled output data showed significant improvement. When a load is applied in a single direction, the output of inter-dimensional coupling in the remaining directions is nearly zero, which indicates that the improved BP neural network model effectively decoupled the parallel six-axis accelerometer.

The weight matrices netiw and netlw and threshold vectors $netb_{1}$ and $netb_{2}$ of the improved BP neural network nonlinear decoupling model input layer and implied layer as well as the implied layer and output layer for the completed training are shown in Equations (7)–(10), respectively. The last three columns of the hidden and output layer weight matrices netlw and threshold vectors $netb_{2}$ are zeros, so they are not listed.

(7) $$netiw=\left[\begin{array}{ccc} \begin{array}{cc} -0.2908 & -0.6785\\ -0.0547 & -0.1550\\ 0.4181 & 1.3622 \end{array} & \begin{array}{cc} 0.0574 & -1.5083\\ -0.0534 & 0.8712\\ 0.0611 & -0.4864 \end{array} & \begin{array}{cc} 1.2566 & -1.1614\\ 0.9643 & 0.4250\\ 1.2950 & -1.5081 \end{array}\\ \begin{array}{cc} 0.6189 & -0.1667\\ -1.5880 & -4.5071\\ -0.9996 & 0.3020 \end{array} & \begin{array}{cc} 0.0635 & 0.0656\\ 1.9444 & 2.1085\\ -0.6531 & -1.1671 \end{array} & \begin{array}{cc} -1.2430 & -1.0546\\ -1.4133 & 2.6327\\ -1.0238 & -0.6456 \end{array}\\ \begin{array}{cc} -0.0636 & -1.2774\\ 0.6202 & -0.2638\\ 3.9390 & -0.6464 \end{array} & \begin{array}{cc} 0.0075 & -1.1045\\ 0.3139 & 0.9316\\ -1.4848 & -0.7248 \end{array} & \begin{array}{cc} 1.0011 & -0.1498\\ 0.8249 & 0.4789\\ 2.1433 & -0.9954 \end{array} \end{array}\right]$$

(8) $$netlw=\left[\begin{array}{c} \begin{array}{cc} \begin{array}{cc} 0.0013 & 0.3455 \end{array} & \begin{array}{cc} -0.0002 & 0.0003 \end{array} \end{array}\\ \begin{array}{cc} \begin{array}{cc} 0.0000 & 0.3124 \end{array} & \begin{array}{cc} -0.0008 & 0.0003 \end{array} \end{array}\\ \begin{array}{cc} \begin{array}{cc} -0.0013 & -0.1348 \end{array} & \begin{array}{cc} 0.0008 & 0.0000 \end{array} \end{array} \end{array}\right.\;\left.\begin{array}{c} \begin{array}{cc} \begin{array}{cc} -0.0001 & 0.0026 \end{array} & \begin{array}{ccc} -0.0001 & 0.0004 & -1.0012 \end{array} \end{array}\\ \begin{array}{cc} \begin{array}{cc} -1.0007 & -0.0011 \end{array} & \begin{array}{ccc} 0.0003 & -0.0068 & 0.0000 \end{array} \end{array}\\ \begin{array}{cc} \begin{array}{cc} 1.0007 & -0.0027 \end{array} & \begin{array}{ccc} 0.0000 & -0.0063 & 1.0012 \end{array} \end{array} \end{array}\right]$$

(9) $${netb}_{1}=\left[\begin{array}{cc} \begin{array}{cc} 1.4547 & 3.5592 \end{array} & \begin{array}{cc} -1.1256 & -1.5181 \end{array} \end{array}\right.\;{\left.\begin{array}{cc} \begin{array}{cc} 2.9313 & -1.3492 \end{array} & \begin{array}{ccc} -1.4636 & 1.8106 & 3.5844 \end{array} \end{array}\right]}^{T}$$

(10) $$netb_{2}={\left[\begin{array}{ccc} -0.3472 & -0.3062 & -0.8616 \end{array}\right]}^{T}$$

(11) $$C_{a}=\left[\begin{array}{ccc} \begin{array}{cc} -0.0095 & 0.0069\\ -0.0096 & 0.0065\\ 0.0218 & -0.0121 \end{array} & \begin{array}{cc} -0.0042 & 0.0003\\ -0.0035 & 0.0007\\ 0.0078 & -0.0017 \end{array} & \begin{array}{cc} 0.0060 & 0.0014\\ 0.0083 & -0.0014\\ -0.1620 & 0.0010 \end{array} \end{array}\right]$$

According to the static calibration experimental data, the Gough–Stewart structure six-axis accelerometer calibration matrix based on the least squares method can be obtained as shown in Equation (11). Selected random static calibration data are decoupled by Equation (7), and the decoupling accuracies of linear calibration decoupling three orthogonal-axis direction acceleration can be obtained as 2.6%, 3.7% and 0.4%.

According to the test results of the nonlinear decoupling model based on the improved BP neural network, the calibrated decoupling error of the acceleration of each axis of the six-axis accelerometer can be obtained, as shown in Table 5.

The decoupling error data presented in Table 5 reveal that, as the calibrated loaded linear acceleration increases, the decoupling accuracy of the *x*-axis linear acceleration gradually decreases, while the decoupling accuracy of the *y*-axis linear acceleration shows a trend of decreasing and then increasing. The decoupling accuracy of the *z*-axis linear acceleration tends to be stable in general. The decoupling accuracies of *x*-, *y*- and *z*-axis acceleration of the six-axis accelerometer after the decoupling of the improved BP neural network model are 0.035%, 0.018% and 0.039%, respectively. Overall, the decoupling ac-curacies remain within 0.05%, representing a significant improvement compared to the linear decoupling method based on the least squares approach.

## 5. Conclusions

In this paper, in view of the limitations of the existing linear decoupling methods for six-axis accelerometers, a nonlinear decoupling study of six-axis accelerometers has been carried out by improving BP neural networks, and the following conclusions have been drawn:

The decoupling model based on the improved BP neural network constructs a good nonlinear mapping relationship between the input and output of the six-axis accelerometer, which solves the problem that the application of linear decoupling methods such as the least squares method to the six-axis accelerometer cannot fully reflect the poor measurement accuracy caused by the nonlinear factors of the sensor system.

The standard BP neural network is topologically optimized using the LM algorithm as well as the gradient descent with momentum, which makes the neural network nonlinear decoupling model have better shock resistance and improves the decoupling accuracy while converging faster.

By comparing the accuracy of the neural network model calibration decoupling and the least squares decoupling results, the six-axis accelerometer decoupling model based on BP neural network is very effective, and the measurement accuracy has been significantly improved, which provides theoretical support for the study of nonlinear decoupling of six-axis accelerometers.

## Figures and Tables

**Table 1:** Six-channel output voltage for *x*-axis calibration experiments.

No.	Standard Value (m/s^2^)	Output Voltage (mV)					
		Channel 1	Channel 2	Channel 3	Channel 4	Channel 5	Channel 6
1	1	129.7	425.5	583.4	457	115.6	592.6
2	2	259.8	853.4	1162.6	955.2	233	1184.6
3	3	385.5	1282.2	1748.4	1446.6	351.9	1782.3
4	4	514.8	1713.6	2337.2	1938	468.8	2383.2
5	5	645	2147.5	2932	2433.5	584	4.99831

**Table 2:** Six-channel output voltage for *y*-axis calibration experiments.

No.	Standard Value (m/s^2^)	Output Voltage (mV)					
		Channel 1	Channel 2	Channel 3	Channel 4	Channel 5	Channel 6
1	1	600.6	399.7	217.5	390.8	591	180.2
2	2	1202.4	796.4	436.8	780.2	1166.2	354.8
3	3	1808.1	1193.7	658.8	1170	1755	533.7
4	4	2416.8	1594.8	887.6	1554.8	2351.6	706.4
5	5	3037	1999.5	1119.5	1939	2952	883

**Table 3:** Six-channel output voltage for *z*-axis calibration experiments.

No.	Standard Value (m/s^2^)	Output Voltage (mV)					
		Channel 1	Channel 2	Channel 3	Channel 4	Channel 5	Channel 6
1	1	383.1	351.6	374.7	373.2	354.2	380.3
2	2	765.6	705.2	750.8	748.4	711.6	755.8
3	3	1146.9	1056.6	1123.2	1121.4	1065.9	1128.9
4	4	1531.6	1413.6	1499.2	1504.4	1426.4	1510.4
5	5	1915.5	1767	1874	1883	1785.5	1879.5

**Table 4:** The decoupled output of the network model.

Input (m/s^2^)	Decoupled Output of *x*-Axis Loading (m/s^2^)			Decoupled Output of *y*-Axis Loading (m/s^2^)			Decoupled Output of *y*-Axis Loading (m/s^2^)		
	*a*_*x*_	*a*_*y*_	*a*_*z*_	*a*_*x*_	*a*_*y*_	*a*_*z*_	*a*_*x*_	*a*_*y*_	*a*_*z*_
1	0.99984	−3.06 × 10^−3^	3.22× 10^−3^	−1.66 × 10^−3^	0.99984	1.71 × 10^−4^	1.21 × 10^−4^	−6.51	0.99961
2	1.99966	−6.40 × 10^−3^	6.73 × 10^−3^	−4.39 × 10^−3^	1.99966	3.8 × 10^−4^	2.24 × 10^−4^	−1.22 × 10^−2^	1.99928
3	2.99944	−9.63 × 10^−3^	1.02 × 10^−2^	−4.73 × 10^−3^	2.99954	5.01 × 10^−4^	3.33 × 10^−4^	−1.85 × 10^−2^	2.99896
4	3.99924	−1.3 × 10^−2^	1.37 × 10^−2^	−6.30 × 10^−3^	3.99940	6.57 × 10^−4^	4.17 × 10^−4^	−2.30 × 10^−2^	3.99867
5	4.99904	−1.66 × 10^−2^	1.75 × 10^−2^	−7.50 × 10^−3^	4.99928	7.86 × 10^−4^	5.21 × 10^−4^	−2.85 × 10^−2^	4.99831
6	5.99883	−2.02 × 10^−2^	2.13 × 10^−2^	−9.41 × 10^−3^	5.99914	9.38 × 10^−4^	6.05 × 10^−4^	−3.32 × 10^−2^	5.99803
7	6.99866	−2.32 × 10^−2^	2.45 × 10^−2^	−1.05 × 10^−2^	6.99910	9.93 × 10^−4^	7.03 × 10^−4^	−3.80 × 10^−2^	6.99765
8	7.99842	−2.92 × 10^−2^	3.07 × 10^−2^	−1.55 × 10^−2^	7.99896	1.17 × 10^−3^	7.97 × 10^−2^	−4.30 × 10^−2^	7.99732
9	8.99801	−4.73 × 10^−2^	4.93 × 10^−2^	−1.87 × 10^−2^	8.99852	1.64 × 10^−3^	8.95 × 10^−2^	−4.74 × 10^−2^	8.99691
10	9.99645	−7.63 × 10^−2^	8.00 × 10^−2^	−1.98 × 10^−2^	9.99819	1.98 × 10^−3^	9.46 × 10^−2^	−4.89 × 10^−2^	9.99655

**Table 5:** Calibration decoupling error for neural network models.

Calibration Acceleration(m/s^2^)	Error of $a_{x}$ (%)	Error of $a_{y}$ (%)	Error of $a_{z}$ (%)
1	0.016	0.015	0.039
2	0.017	0.017	0.036
3	0.018	0.015	0.035
4	0.018	0.015	0.033
5	0.019	0014	0.034
6	0.019	0.014	0.033
7	0.019	0.013	0.034
8	0.019	0.013	0.033
9	0.022	0.016	0.034
10	0.035	0.018	0.034

## Data Availability

The data set is within the article.
